# Therapeutic potential activity of quercetin complexes against *Streptococcus pneumoniae*

**DOI:** 10.1038/s41598-024-62782-w

**Published:** 2024-06-05

**Authors:** Mohamed E. Osman, Amany A. Abo-Elnasr, Eslam T. Mohamed

**Affiliations:** Botany and Microbiology Department, Faculty of Science, Helwan University, Ain Helwan, Cairo, 11795 Egypt

**Keywords:** *Streptococcus pneumoniae*, Quercetin, Metal complexes, Synergism, Virulence inhibition, Biofilm, Microbiology, Medical research

## Abstract

This study investigates quercetin complexes as potential synergistic agents against the important respiratory pathogen *Streptococcus pneumoniae*. Six quercetin complexes (QCX1–6) were synthesized by reacting quercetin with various metal salts and boronic acids and characterized using FTIR spectroscopy. Their antibacterial activity alone and in synergism with antibiotics was evaluated against *S. pneumoniae* ATCC 49619 using disc diffusion screening, broth microdilution MIC determination, and checkerboard assays. Complexes QCX-3 and QCX-4 demonstrated synergy when combined with levofloxacin via fractional inhibitory concentration indices ≤ 0.5 as confirmed by time-kill kinetics. Molecular docking elucidated interactions of these combinations with virulence enzymes sortase A and sialidase. A biofilm inhibition assay found the synergistic combinations more potently reduced biofilm formation versus monotherapy. Additionally, gene–gene interaction networks, biological activity predictions and in-silico toxicity profiling provided insights into potential mechanisms of action and safety.

## Introduction

The escalating threat of antibiotic resistance in pathogens like *Streptococcus pneumoniae* necessitates the exploration of novel therapeutic strategies. While existing pneumococcal vaccines and antibiotics have proven effective, the emergence of non-vaccine serotypes and increasing resistance demand innovative solutions. This study delves into the potential of quercetin, a natural flavonoid with known antimicrobial properties, as a potential weapon in the fight against *S. pneumoniae* infections^1–8^.

Quercetin's ability to disrupt bacterial membranes, inhibit efflux pumps, and interfere with essential cellular processes has demonstrated its effectiveness against various pathogens. However, the synergistic potential of quercetin when combined with conventional antibiotics against *S. pneumoniae* remains largely unexplored. This study aims to bridge this gap by evaluating the combined effects of quercetin and its metal complexes with antibiotics on S. pneumoniae growth and virulence^9–12^.

The investigation goes beyond simply observing synergistic effects; it delves into the mechanisms underlying this synergy. Specifically, the study focuses on the inhibition of crucial virulence factors like sialidase NanB and sortase A. These factors play key roles in *S. pneumoniae* colonization and biofilm formation, contributing to its pathogenesis. By targeting multiple pathways involved in infection, this approach offers a promising avenue to combat antibiotic resistance and improve treatment outcomes^13–15^.

Furthermore, the study extends beyond free quercetin to explore the enhanced antimicrobial activity of its metal complexes. These complexes have demonstrated superior inhibitory effects on bacterial efflux pumps and other cellular processes compared to quercetin alone. This exploration holds significant potential for developing more potent therapeutic agents against *S. pneumoniae* and overcoming the challenges posed by antibiotic resistance.

## Material and methods

### Production, extraction, and purification of extracellular bioactive secondary metabolites by liquid-state fermentation

Metabolites of Streptomyces isolate was produced using submerged culture of *Streptomyces thinghirensis* WAE1 (accession no. ON584359.1)^16^. Five percent inoculum size of culture was inoculated in glucose soybean meal broth medium (glucose (10 g/L), soybean meal (14 g/L), CaCO_3_ (1 g/L), NaCl (16 g/L), pH of 9) for 8 days at 35 °C^17^. After the incubation period, the cultures were collected and filtered with sterilized filter paper. The clear filtrate was collected and subjected to extraction using different organic solvents (acetone, chloroform, ethyl acetate, methanol, n-butanol, and n-hexane). The extracts were then dried in a rotary evaporator at 40 °C to remove the solvents. Their antibacterial activity against *S. pneumoniae* ATCC 49,619 was then analyzed using the disc diffusion method (twenty microgram of each extract/disc)^18^.

### Purification of the crude extract by TLC

The ethyl acetate crude extract was initially purified using thin-layer chromatography (TLC). A pre-coated silica gel aluminum TLC plate was used as the stationary phase, with a chloroform: methanol mobile phase in a 9:1 ratio. A small amount of sample was applied to the starting line of the plate using a capillary tube. The plate was then placed in a developing chamber containing the mobile phase solvents. After 30 min of incubation at room temperature, the plate was removed and allowed to dry. Next, the fractions separated on the TLC plate were tested for antimicrobial activity using the disc diffusion assay. The fraction displaying the clearest zone of inhibition was selected for further purification by column chromatography for facilitating purification and identification of the antimicrobial compound^19^.

### Purification by column chromatography

The crude extract was subjected to column chromatography to isolate bioactive components. The extract was applied to a silica gel column as the stationary phase. A mobile phase of chloroform and methanol in a 9:1 ratio was used to elute different fractions. These fractions were then tested for antibacterial activity against Streptococcus pneumoniae using the disc diffusion assay. The fraction producing the largest zone of inhibition was selected as the most promising for further characterization. In addition, fractions demonstrating identical retention factors on TLC analysis were combined to provide enriched samples^20^.

### Identification of the produced antimicrobial agent

The active metabolite was identified by analyzing it using UV spectrophotometry, HPLC, FTIR spectrophotometry, ^1^H and ^13^C NMR, mass spectrometry, solubility and melting point tests, and elemental analysis. The UV–visible absorption spectra were recorded using a spectrophotometer in the UV–visible region (200–800 nm), and the HPLC assay was performed using a Shimadzu LC-20 apparatus. Spectral analysis FTIR using Jasco FT-IR spectrophotometer 4100, and the NMR spectrum was recorded using a Bruker Biospin AVANCE II 300. Mass spectra were obtained using a GCMS-QP2010 SE apparatus, and the solubility and melting point was determined with a digital Stuart SMP3 electric melting point apparatus. The elemental analysis of C, H, N, O, and S was conducted using a Vario EL III CHNOS elemental analyzer.

### Synthesis of quercetin complex (QCX) compounds and spectroscopic Measurements

Three quercetin metal complexes (QCX-1, QCX-2, QCX-3) were synthesized by mixing quercetin as the ligand with FeSO_4_·7H_2_O, Cu(CH_3_COO)_2_, and NiCl_2_ in a 2:1 molar ratio of ligand to metal salt. The reactions were carried out in methanol and water at room temperature for 3 h, after which triethylamine was added to induce precipitation. The products were isolated after 2 days of refrigeration and drying. Similarly, three quercetin boron complexes (QCX-4, QCX-5, QCX-6) were synthesized. For each reaction, quercetin was dissolved in tetrahydrofuran and refluxed, then an equimolar amount of 1,4-phenyl diboronic acid, 6-methoxy-3-pyridinylboronic acid, or 6-methoxynaphthalene boronic acid respectively was added to the mixture. Reflux continued for 24 h to yield the solid boron complexes, which were purified by precipitation, washing, and drying. Fourier-transform infrared (FTIR) spectroscopy was conducted to analyze the synthesized QCX compounds^12,21,22^.

### Antibiotic susceptibility testing and MIC of the synthesized compounds

The susceptibility of *S. pneumoniae* ATCC 49619 to various antibiotics was initially screened using the agar disc diffusion assay. Discs containing penicillin, cefalexin, tetracycline, kanamycin, and levofloxacin were placed on inoculated nutrient agar plates and inhibition zone diameters were measured after 24 h. Compounds exhibiting activity, including the quercetin metabolite and synthesized QCX complexes, were further evaluated for minimum inhibitory concentration (MIC) via broth microdilution methods. For the latter, a range of two-fold dilutions from 0.125 to 512 μg/mL of each test agent were prepared in Muller Hinton broth inoculated with 10^5^ CFU/mL of bacteria according to CLSI guidelines^23^. The MIC was defined as the lowest concentration that prevented visible growth after 24 h of incubation at 37 °C.

### Drug synergy experiments and fractional inhibitory concentration (FIC) and in-vitro time kill assay

The synergistic effects between the most potent antibiotics and QCX compounds were evaluated using the checkerboard microdilution method. Two-fold serial dilutions of each antibiotic and QCX compound alone and in combination were prepared in 96-well plates containing Muller Hinton broth and inoculated with 10^5^ CFU/mL of bacteria. The concentration range tested for both the antibiotics and the QCX compounds in the checkerboard method spanned from the minimum inhibitory concentration (MIC) of each agent down to 1/8 of the MIC, with two-fold dilutions. This concentration range allowed for the determination of the fractional inhibitory concentrations (FICs) and the calculation of the fractional inhibitory concentration index (FIC_i_) to assess the nature of the interactions between the antibiotics and the QCX compounds. The plates were incubated at 37 °C for 24 h and observed for visible growth. Synergy was defined as FIC_i_ ≤ 0.5, an additive effect as > 0.5 to ≤ 1, and antagonism as FIC_i_ > 1^24,25^.

$${\text{FIC}}_{{\text{i}}} = {\text{FIC}}_{{\text{A}}} + {\text{FIC}}_{{\text{B}}}$$

where,

$${\text{FIC}}_{{\text{A}}} = \frac{{{\text{MIC }}\;\left( {{\text{Drug}}\;{\text{A}}} \right)\;{\text{in}}\;{\text{combination}}}}{{{\text{MIC }}\;\left( {{\text{Drug}}\;{\text{A}}} \right)\;{\text{alone}}}};\quad {\text{FIC}}_{{\text{B}}} = \frac{{{\text{MIC}}\;{ }\left( {{\text{Drug}}\;{\text{B}}} \right)\;{\text{in}}\;{\text{combination}}}}{{{\text{MIC}}\;{ }\left( {{\text{Drug}}\;{\text{ B}}} \right)\;{\text{alone}}}}$$

The time-kill assay was carried out to assess the bactericidal kinetics of the most synergistic antibiotic-QCX combinations identified previously. The combination with the lowest FIC_i_ was selected for this analysis. The selected combinations were inoculated with the bacterial culture at an initial density of 10^5^ CFU/mL, then incubated at 37 °C. The optical density at 600 nm was measured spectrophotometrically at regular intervals from 0 to 24 h to monitor bacterial growth over an extended period. This time-kill study provided kinetic data on the rate and extent of bacterial killing by the synergistic antibiotic-QCX combination^26^.

### Effect of selected drug combinations on biofilm formation and cell viability

The ability of antibiotic-QCX combinations to inhibit biofilm formation was tested using a microtiter plate assay. Bacterial strains were incubated for 24 h at 37 °C in 96-well plates containing tryptic soy broth with two-fold serial dilutions of each test agent alone and in combination. After incubation, non-adherent planktonic cells were removed by washing with distilled water. Adherent biofilms were fixed with methanol. The biofilms were then stained with 0.1% crystal violet for 15 min, washed with distilled water, and dissolved in 33% acetic acid. The optical density of the dissolved biofilms was measured at 585 nm. The percentage inhibition of biofilm formation for each treatment was calculated relative to the optical density of the untreated control, using the formula^27^:

$${\text{Percentage}}\;{\text{inhibition}}\;\left( \% \right) = \left[ {\left( {{\text{OD}}\;{\text{control}} - {\text{OD}}\;{\text{treatment}}} \right)/{\text{OD}}\;{\text{control}}} \right] \times {1}00.$$

### In-Silico docking study

Molecular docking was performed to model the interactions between levofloxacin, QCX-3, QCX-4 and the active sites of SrtA and NanB sialidase enzymes. The crystal structures of these proteins (PDB IDs 1T2W and 2VW0) were prepared using protein preparation in Schrodinger, removing water and other small molecules. The binding pockets were characterized using SiteMap. Ligands were generated from PubChem data (https://pubchem.ncbi.nlm.nih.gov/), preprocessed with LigPrep, and minimized using Maestro. Extra precision (XP) Glide docking was carried out to predict stable binding orientations and energies of ligands within the active sites. The default rigorous docking protocol was employed to systematically explore binding poses and score ligand–protein interactions using Glide's optimized empirical scoring function^28^.

### Gene–gene interaction network analysis

The GeneMANIA platform (http://genemania.org) was used to predict genetic and physical interactions between query genes. GeneMANIA integrates multiple genomics and proteomics datasets to functionally characterize query genes and prioritize candidate genes. The query genes input were glmM (SPD_0186), which encodes phosphoglucosamine mutase involved in peptidoglycan synthesis, nanA (SPD_1499) which encodes neuraminidase A possessing sialidase activity, and sodA (SPD_1207) which encodes superoxide dismutase, an antioxidant defense modulated by NanB. GeneMANIA analyzed functional association data, including protein and genetic interactions, pathway data, co-expression, co-localization and protein domain similarity, to build an interaction network for the query genes. This allowed prediction of relationships between the query genes based on the diverse genomics and interaction datasets integrated within the GeneMANIA platform^29^.

### Biological activities

The Way2Drug platform (PASS online, http://way2drug.com/PassOnline/) was used to predict the biological activities of levofloxacin, quercetin, QCX-3, and QCX-4 based on molecule structures. PASS Online was used to investigate these compounds' mechanisms of action and potential therapeutic effects. The study evaluated the probability of therapeutic effects such as antimicrobial activities for the compounds after predicting their biological targets^30^.

### Computational toxicity (ADMET) prediction

The ADMET properties of levofloxacin, quercetin, QCX-3 and QCX-4 were evaluated in-silico using AdmetSAR (version 1.0; School of Pharmacy, Shanghai Jiao Tong University, China). Parameters examined included human intestinal absorption, Caco-2 cell permeability as measures of absorption, solubility, and subcellular localization. Interactions with major cytochrome P450 isozymes (CYP1A2, 2C9, 2D6, 3A4) involved in drug metabolism were also predicted. Toxicological factors assessed were AMES mutagenicity, biodegradability, carcinogenicity, acute oral and rat acute toxicity. AdmetSAR (admetlab 1.0, http://lmmd.ecust.edu.cn/admetsar1) utilizes machine learning algorithms to predict ADMET profiles based on chemical structure data, providing insights into the compounds’ potential mechanisms of action, metabolism and toxicity risks in-silico^30^.

### Statistical analysis

Data are presented as mean values and standard deviations (SD), alongside synergy analysis and visualization of drug combinations using Combenefit software^31^.

## Results

### Extraction of antimicrobial compound

#### Antimicrobial of crude extract of WAE1 isolate

**Figure 1:**
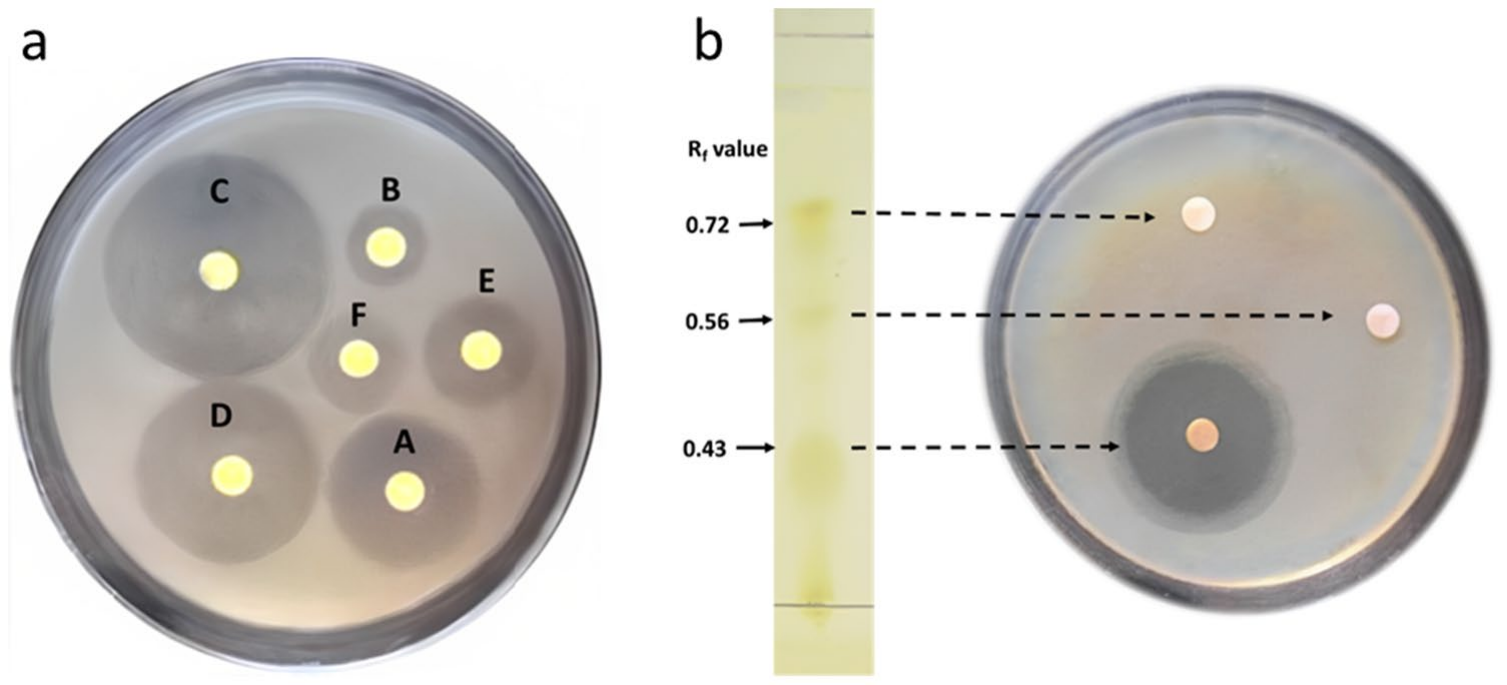
(**a**) Antibacterial inhibition zone diameters of crud extract of *S. thinghirensis* WAE1 using different solvents (acetone (A), chloroform (B), ethyl acetate (C), methanol (D), n-butanol (E), and n-hexane (F)), showing the largest zone formed by ethyl acetate extract and (**b**) Antibacterial inhibition zones against *S. pneumoniae* for fractions of the *S. thinghirensis* WAE1 crude extract separated by TLC, showing activity only for the fraction at R_f_ 0.43.

The antimicrobial compound was extracted from *S. thinghirensis* WAE1 under optimized conditions and tested for antibacterial activity against *S. pneumoniae* using different organic solvents. Ethyl acetate extraction yielded the highest amount of crude extract (840 mg) and produced the largest zone of inhibition at 34.85 ± 0.71 mm. Methanol and acetone extractions also demonstrated moderate activity at 27.45 ± 0.11 mm and 21.41 ± 0.15 mm, respectively. In contrast, extraction with chloroform produced the lowest yield (72 mg) and smallest zone (11.72 ± 0.45 mm), indicating it was the least effective solvent for recovering antibacterial components. n-Butanol and n-hexane extracts displayed intermediate effects (Supplementary Table S1 and Fig. 1a).

#### Thin layer chromatography

**Figure 2:**
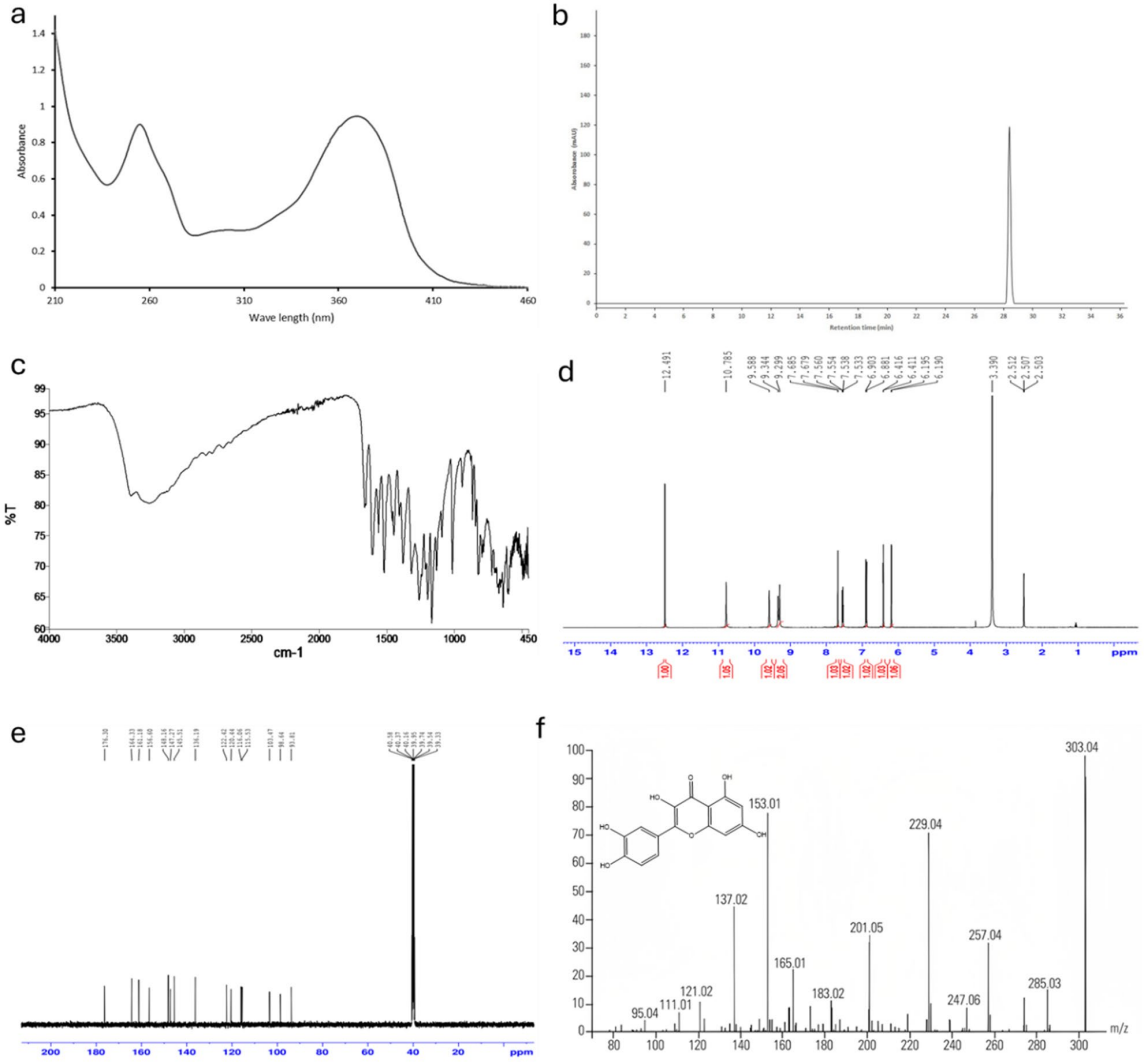
Analytical characterization of the active metabolite (a. UV spectrum, b. HPLC spectrum, c. FTIR spectrum, d. 1H NMR (400 MHz) spectrum, e. 13C NMR spectrum and f. mass spectrum).Fig. 4. The structures and FTIR spectra of QCX-1 (**a**), QCX-2 (**b**), QCX-3 (**c**), QCX-4 (**d**), QCX-5 (**e**) and QCX-6 (**f**) show characteristic absorption bands indicative of functional groups present in the compounds.

Thin layer chromatography (TLC) was used to separate metabolites in the crude extract. As shown in Supplementary Table S2 and Fig. 2b, three spots were detected on the TLC plate using a chloroform:methanol (9:1) mobile phase with Rf values of 0.43, 0.56, and 0.72. The antibacterial activity of each spot against *S. pneumoniae* was then determined using the disc diffusion assay. Only the spot with an R_f_ of 0.43 exhibited a zone of inhibition, measuring 26.32 mm. The spots at R_f_ 0.56 and 0.72 did not demonstrate any activity. This suggests that bioactive compounds responsible for the antimicrobial effects are concentrated in the 0.43 R_f_ fraction separated by this solvent system.

#### Purification by silica gel column

Silica gel column chromatography was employed to further purify the bioactive fraction isolated previously by TLC (Rf 0.43). Gradient elution using chloroform: methanol (9:1) yielded 14 collected fractions that were analyzed by TLC and tested for antimicrobial activity against *S. pneumoniae* via disc diffusion assay. As shown in Table 3, fractions 4–12 demonstrated zones of inhibition ranging from 8.1 to 21.9 mm, while also co-migrating with an Rf of 0.43 on TLC, indicating those fractions contained the purified active compound. The remaining fractions 1–3 and 13–14 did not inhibit bacterial growth. These results suggest the potent metabolite was successfully enriched in fractions 4–12 through column separation (Supplementary Table S3).

#### Identification of the produced active extracted metabolite

The active metabolite fraction has been investigated using a UV spectrophotometer at range (100–500 nm), showing two specific UV absorption peaks at 257 and 370 nm. HPLC assay revealed a single peak at retention time 28.85 min, ensuring the purity of the active compound. FTIR Spectrum of the active metabolite indicated the presence of functional groups such as O–H stretching vibration of phenol, C=O aryl ketonic stretch, C–C aromatic ring stretch, C=O aromatic stretch, C=C aromatic stretch, O–H bending of phenols, C–H bond in aromatic hydrocarbon, C–O stretch of aryl ether, C–O stretch of phenol, C–CO–C stretch and bending in ketone, and C–H bending of aromatic hydrocarbons. The ^1^H NMR spectrum displayed characteristic hydrogen peaks at δ, ppm: 12.49 (s,^1^H, OH-5), 10.78 (s, ^1^H, OH-7), 9.65 (s, ^1^H, OH-4'), 9.34 (s, ^1^H,OH-3), 7.67 (d, J = 2.2 Hz, ^1^H, H-2'), 7.53 (d, J = 8.3 Hz, ^1^H, H-6'), 6.90 (d, J = 8.4 Hz, ^1^H, H-5'), 6.41 (d, J = 2.2 Hz, ^1^H, H-8), 6.19 (d, J = 2.2 Hz, ^1^H, H-6). The ^13^C NMR spectrum showed relevant peaks at (ppm): 176.30 (C-4), 164.33 (C-7), 161.18 (C-5), 156.60 (C-9), 148.16 (C-4'), 147.27 (C-2), 145.51 (C-3'), 136.19 (C-3), 122.42 (C-1'), 120.44 (C-6'), 116.06 (C-5'), 115.53 (C-2'), 103.47 (C-10), 98.64 (C-6), 93.81 (C-8). Elementary analysis (%) of the active metabolite shows that C = 59.55%, H = 3.30%, O = 37.05%, N = 0.00% and S = 0.00%. These analyses suggested an empirical formula of C_15_H_10_O_7_ and consequently the chemical structure formula is 2-(3,4-dihydroxyphenyl)-3,5,7-trihydroxychromen-4-one which is known as quercetin. The antibacterial compound was obtained as a yellow powder with a melting temperature of 315–316 °C. It is soluble in dimethyl sulfoxide (DMSO), methanol, ethanol and acetone (Fig. 2).

#### Structural investigation of QCX compounds

**Figure 3:**
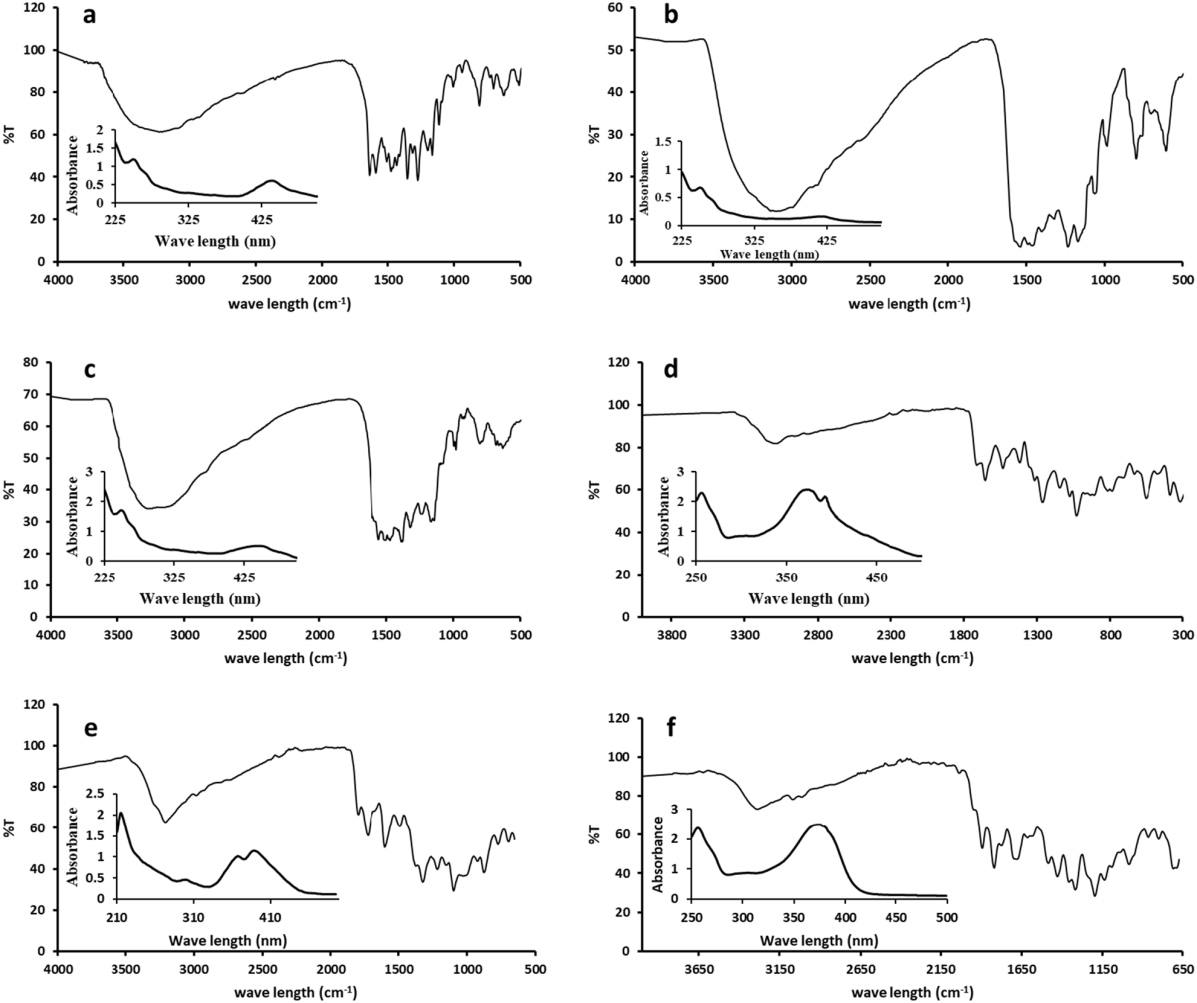
The FTIR spectra and UV-spectra of QCX-1 (**a**), QCX-2 (**b**), QCX-3 (**c**), QCX-4 (**d**), QCX-5 (**e**) and QCX-6 (**f**) show characteristic absorption bands indicative of functional groups present in the compounds.

The infrared spectroscopy data provides valuable information about the functional groups present in compounds QCX-1 to QCX-6. For QCX-1 to QCX-3, the presence of absorption bands around 3200–3100 cm^−1^ indicates the presence of O–H groups. The bands around 1300–1400 cm-1 correspond to C–OH bending. The peaks in the 1280–1220 cm^−1^ region relate to C–O–C stretching vibrations. Additionally, the bands near 1600–1650 cm^−1^ suggest the presence of C=O groups. For QCX-4 to QCX-6, the peaks around 1600–1700 cm^−1^ again indicate C=O stretches. The absorptions in the 1500–1600 cm^−1^ range are characteristic of C=C stretches in aromatic rings. These compounds also show fingerprints of B-C, B-Ph, Ar–O and B–O bonds in the expected regions (Fig. 3).

The comprehensive UV–vis spectroscopic analysis revealed the distinct absorption profiles of the quercetin ligand and its various metal complexes, providing valuable insights into the coordination environments. The quercetin spectrum exhibited two characteristic bands: a band at 370 nm (band I) corresponding to the cinnamoyl system of the B-ring, and a band at 257 nm (band II) associated with the benzoyl system of the A-ring. Upon complexation, the absorption spectra of the synthesized compounds, including QCX-1, QCX-2, and QCX-3, displayed distinct bathochromic shifts, confirming the formation of metal–ligand complexes, with the band I positions shifting to 442 nm, 420 nm, and 449 nm, respectively, and the band II positions remaining relatively consistent at 256 nm, suggesting the preferential involvement of the cinnamoyl system in the coordination. Complementing these findings, the UV–vis spectra of the other metal complexes, QCX-4 (254 nm, 305 nm, 375 nm, 392 nm), QCX-5 (217–302 nm, 369–390 nm), and QCX-6 (255–274 nm, 306–371 nm), exhibited characteristic absorption bands corresponding to the transitions within the benzene rings and the free hydroxyl groups, providing a comprehensive understanding of the structural features and coordination modes across the series of metal-quercetin complexes (Fig. 3).

#### Antibiotic susceptibility testing and MIC of the antimicrobial compounds

The susceptibility of *S. pneumoniae* ATCC 49619 strain to various antibiotics was estimated. As shown in Supplementary Table S4, the strain demonstrated sensitivity to penicillin (β-lactam), tetracycline (tetracycline), and levofloxacin (fluoroquinolone), but resistance to cefalexin (cephalosporin) and kanamycin (aminoglycoside). The MICs of the susceptible antibiotics, quercetin, and QCX compounds against the strain are summarized in Supplementary Table S5. Penicillin, tetracycline, and levofloxacin had MIC values of 4, 8, and 2 μg/mL, respectively. Quercetin exhibited an MIC of 128 μg/mL. Among the QCX compounds tested, QCX-4 showed the greatest inhibitory activity with an MIC of 1 μg/mL, followed by QCX-3 and QCX-5 with MICs of 2 μg/mL.

#### Determination of fractional inhibitory concentrations (FIC) and In-vitro time kill assay

**Figure 4:**
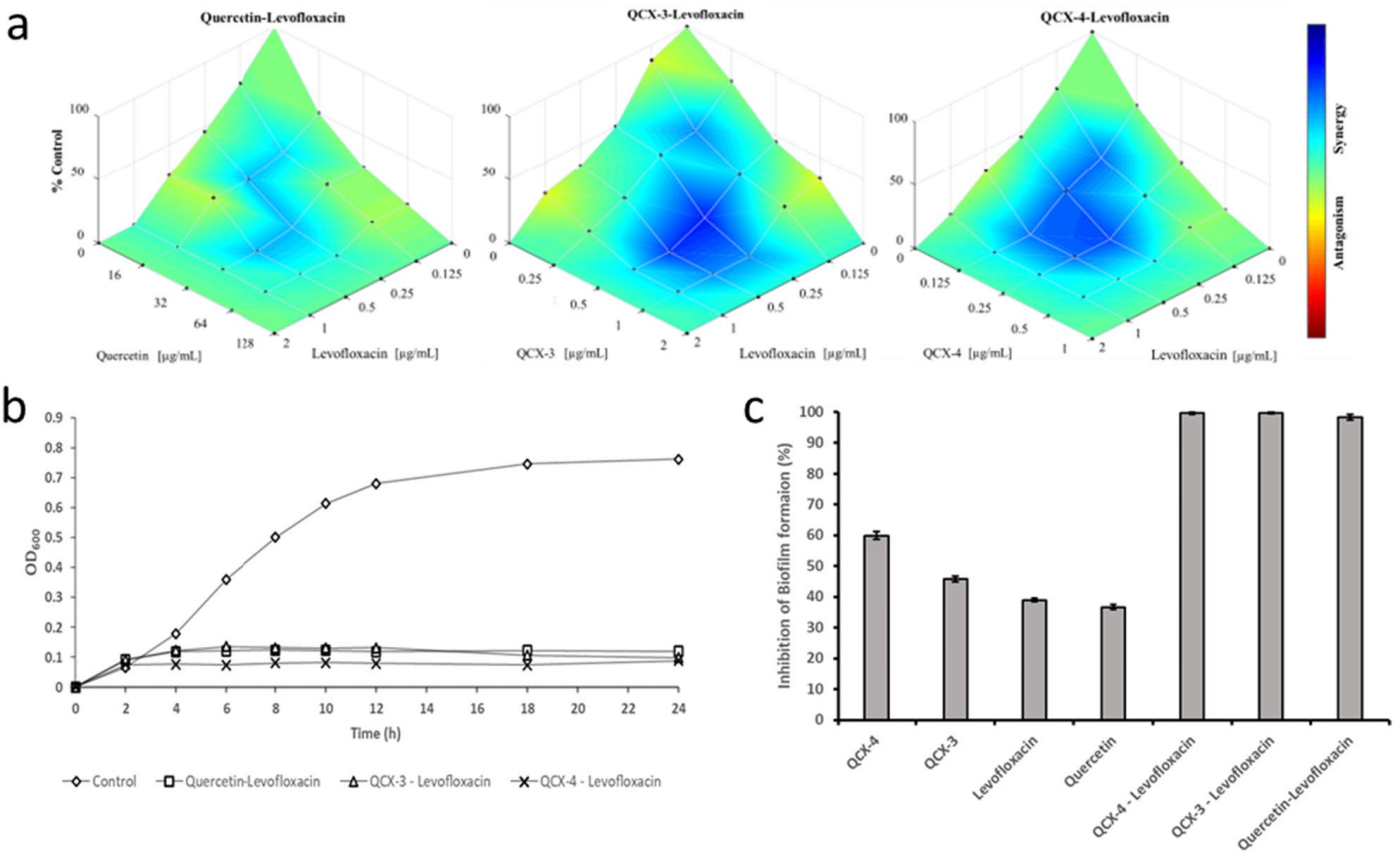
(**a**) Combenefit mapped surface plot visually depicting the synergistic potential between QCX compounds and levofloxacin antibiotic against *S. pneumoniae*. The heat map was generated based on OD600 values after 24 h incubation across a minimum Inhibitory concentration (MIC)-guided concentration matrix, ranging from full MIC down to 1/8 MIC in two-fold serial dilutions for both agent types. Deeper blues illustrate where combinations yielded enhanced reduction beyond individual effects (synergy), while fading yellows and reds indicate no interaction between agents, (**b**) Time-kill assay data showing the growth pattern of *S. pneumoniae* treated with different combinations during 24 h, and (**c**) Effect of different combinations at lowest FIC_i_ on percentage of inhibition of biofilm formation.

The fractional inhibitory concentrations (FICs) and in-vitro time kill assay were performed to determine the nature of interactions between quercetin, QCX compounds, and levofloxacin against *S. pneumoniae*. The FIC index values presented in Table 1 showed that the combinations of quercetin-levofloxacin (FIC_i_ = 0.5), QCX-3—levofloxacin (FIC_i_ = 0.5), and QCX-4—levofloxacin (FIC_i_ = 0.375) exhibited synergism against the bacterial strain. This was further validated by the Combenefit plots generated from the optical density values measured after 24 h of treatment (Fig. 4a), which demonstrated effectively reduction in bacterial growth at the lowest synergistic concentrations. Additionally, the time-kill assay revealed that these combination treatments were able to suppress the growth of *S. pneumoniae* over 24 h (Fig. 4b).

**Table 1:** Fractional inhibitory concentration index (FIC_i_) values for the synergistic different combinations against *S. pneumoniae*.

Antibiotic combinations	Quercetin (Q)—levofloxacin (L)	QCX-3—levofloxacin (L)	QCX-4—levofloxacin (L)
FIC_i_ (FIC_A_: FIC_B_)	0.5 (1/4 Q: 1/4 L)	0.5 (1/4 QCX-3: 1/4 L)	0.375 (1/4 QCX-4: 1/8 L)

#### Effect of selected drug combinations on biofilm formation and cell viability

The biofilm formation was inhibited effectively with drug combinations tested at the lowest FIC_i_ compared to their respective MIC dose (Fig. 4c).

#### In-silico docking studies

**Figure 5:**
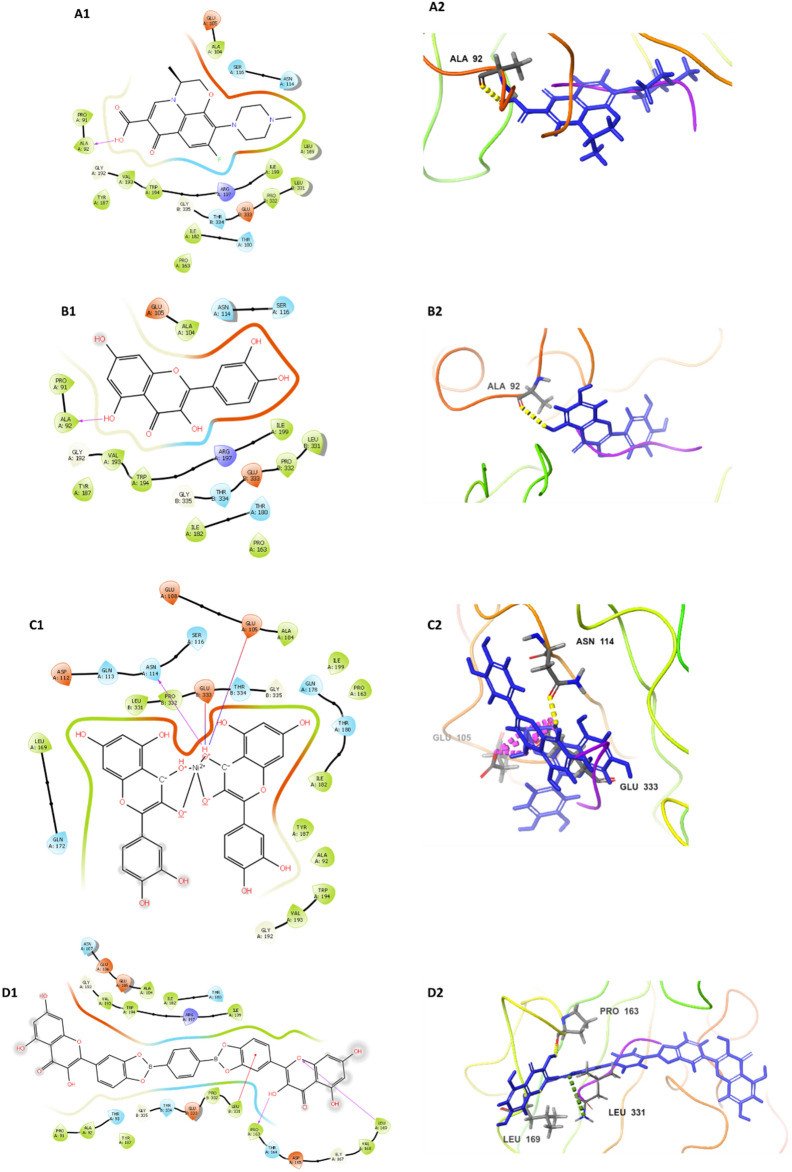
Docking affinity of (**A**) Levofloxacin, (**B**) Quercetin, (**C**) QCX-3 and (**D**) QCX-4 With *S. pneumoniae* SrtA.

**Figure 6:**
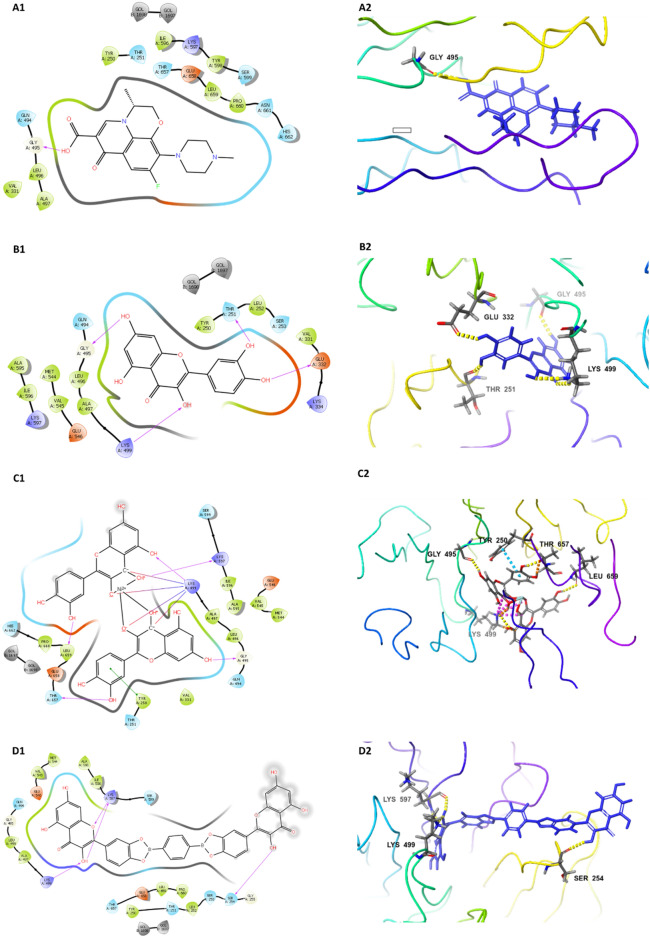
Docking affinity of (**A**) Levofloxacin, (**B**) Quercetin, (**C**) QCX-3 and (**D**) QCX-4 With *S. pneumoniae* sialidase NanB.

The in silico docking analysis revealed that among the studied compounds, QCX-3 and QCX-4 showed highest binding affinity towards SrtA and sialidase NanB as evidenced by their lowest docking scores (− 7.015 kcal/mol and − 7.230 kcal/mol for SrtA, − 7.850 kcal/mol and − 7.295 kcal/mol for NanB respectively). Both the compounds formed multiple hydrogen bond and salt bridge interactions with the protein active sites. In contrast, levofloxacin and quercetin exhibited moderate binding as suggested by their higher docking scores. The results indicate that QCX-3 and QCX-4 may serve as potential lead molecules against SrtA and sialidase NanB (Table 2, Figs. 5 and 6).

**Table 2:** In-silico docking study of studied compounds and crystallographic structure of SrtA and sialidase NanB of *S. pneumoniae*.

Protein	Compound (PubChem ID)	Docking score (kcal/mol)	H-bond	Salt bridge	Pi-cation bond	Pi–pi stacking bond
SrtA	QCX-4	−7.230	PRO A:163	N/A	LEU B:331	N/A
			LEU A:169			
	QCX-3	−7.015	ASN A:114	GLU A:105	N/A	N/A
				GLU B:333		
	Levofloxacin (5280343)	−5.810	ALA A:92	N/A	N/A	N/A
	Quercetin (5280343)	−5.639	ALA A:92	N/A	N/A	N/A
Sialidase NanB	QCX-3	−7.850	GLY A:495	LYS A: 499	N/A	TYR A:250
			LYS A:499			
			LYS A:597			
			LEU A:659			
	QCX-4	−7.295	SER A:254	N/A	N/A	N/A
			LYS A:499			
			LYS A:597			
	Levofloxacin (5280343)	−6.534	GLY A:495	N/A	N/A	N/A
	Quercetin (5280343)	−5.403	THR A:251	N/A	N/A	N/A
			GLU A:332			
			GLY A:495			
			GLY A:499			

*N/A* not present.

#### Gene–gene interaction network analysis

**Figure 7:**
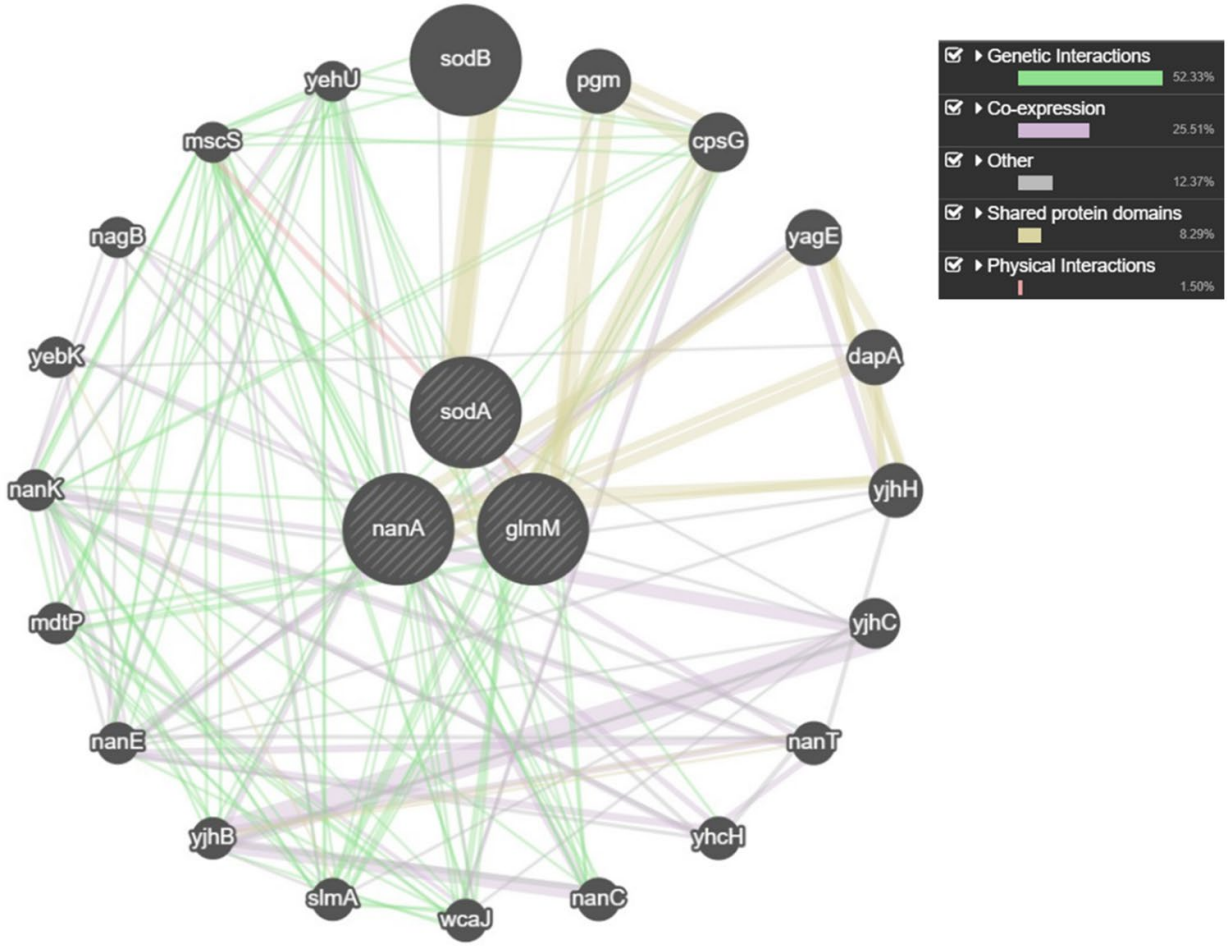
GeneMANIA analysis predicted numerous genetic and physical interactions between query genes glmM, nanA, and sodA, with an interactive network highlighting connections between candidate genes.

The GeneMANIA analysis predicted numerous genetic and physical interactions between the query genes glmM, nanA, and sodA based on different network datasets. The Babu genetic interactions networks provided the most connections, particularly those related to cell envelope biogenesis. The Faith co-expression network also linked the genes based on microarray expression data. Additional evidence came from shared protein domains and physical interaction datasets from Hu and Rajgopala. Many associated genes involved in cell envelope biogenesis were connected, such as sodB, pgm, cpsG. Oxidative stress response genes like sodB also scored highly. Transporters like nanT and channels like mscS ranked amongst the top predictions. Other genes involved in sialic acid metabolism or from the same operons and pathogenicity islands as the queries also showed strong connections (Fig. 7).

#### Biological activity

The biological activities of levofloxacin, quercetin, QCX-3, and QCX-4 were predicted using the Way2Drug platform. For levofloxacin, high probability targets included inhibition of enzymes involved in DNA processing and synthesis, indicating potential antimicrobial. Quercetin was predicted to strongly inhibit (> 0.9 probability) important bacterial functions like membrane integrity, stress responses, and antioxidant defenses, as well as metabolism enzymes, hypoxia response, oxidative stress, and human peroxidase targets, suggesting antibacterial and anti-inflammatory mechanisms. QCX-3's highest probability target was inhibiting *S. pneumoniae* growth, with membrane integrity, kinase inhibition, and effects on bacterial enzymes/metabolism also highly predicted, supporting antibacterial activity. QCX-4 showed strong predicted inhibition of histidine kinases and fatty acid synthase, disrupting signaling and membrane biosynthesis in bacteria. Broad kinase inhibition against regulatory proteins, inhibition of membrane integrity and peroxidase activity (Table 3).

**Table 3:** Biological activities of the selected compounds (PASS online, http://way2drug.com/PassOnline/).

Biological activities (Pa %)	Levofloxacin	Quercetin	QCX-3	QCX-4
Topoisomerase 11 inhibitor	0.967	**–**	–	–
RELA expression inhibitor	0.804	0.813	–	–
HMOXI expression enhancer	0.773	–	–	–
HIFIA expression inhibitor	–	0.919	–	–
Membrane permeability enhancer	–	0.945	0.945	0.925
Membrane integrity antagonist	–	–	0.945	–
Membrane integrity agonist	–	0.945	–	0.945
Peroxidase inhibitor	–	0.807	–	0.825
Anti-inflammatory	–	0.837	–	–
Histidine kinases	–	–	0.929	0.842
kinase inhibition	–	–	0.945	0.929
Fatty acid synthase	–	–	–	0.827

–: no values, Pa: probability of activation.

#### Computational toxicity (ADMET) prediction

The predicted toxicity profiles of levofloxacin, quercetin, QCX-3, and QCX-4 were evaluated in-silico using AdmetSAR. Levofloxacin was predicted to be highly absorbed intestinally, localize to lysosomes, and exhibit low CYP interactions, indicating potential mechanisms of action, while toxicity risks appeared moderate based on assays. Quercetin was predicted to be absorbed intestinally, localize to mitochondria, and potentially inhibit certain CYPs, again indicating mechanisms of action, while toxicity risks appeared low. QCX3 showed favorable predictions of intestinal, mitochondrial localization, and low metabolism by major CYP isozymes, with low predicted toxicity risks. QCX-4 also showed favorable permeability predictions and mitochondrial localization, with potentially low CYP inhibition and metabolism overall. Toxicity risks for QCX-4 were low based on several assays (Table 4).

**Table 4:** ADMET properties for levofloxacin, quercetin, QCX-3, and QCX-4 (Admetlab, http://lmmd.ecust.edu.cn/admetsar1).

Variables	Levofloxacin		Quercetin		QCX-3		QCX-4	
	Results	Prob	Results	Prob	Results	Prob	Results	Prob
Absorption Human Intestinal Absorption	HIA +	0.9545	HIA +	0.9650	HIA +	0.6840	HIA +	0.9790
Caco-2 Permeability	Cac02 +	0.8867	Caco2–	0.8957	Caco2–	0.6522	Caco2–	0.7743
Aqueous solubility [Logs] Distribution	Moderately soluble	−3.5105	Moderately soluble	−2.9994	Moderately soluble	−3.6629	Moderately soluble	−3.4333
Subcellular localization	Lysosome	0.7248	Mitochondria	0.5892	Mitochondria	0.6827	Mitochondria	0.7119
Metabolism								
CYP450 2C9 Substrate	Non-substrate	84.7	Non-substrate	0.7898	Non-substrate	0.8029	Non-substrate	0.7506
CYP450 2D6 Substrate	Non-substrate	0.8468	Non-substrate	0.9116	Non-substrate	0.8317	Non-substrate	0.8623
CYP450 3A4 Substrate	Non-substrate	0.9116	Non-substrate	0.6530	Non-substrate	0.5186	Non-substrate	0.6149
CYP450 IA2 Inhibitor	Non-substrate	0.6386	Inhibitor	0.9106	Non-inhibitor	0.5520	Inhibitor	0.5573
CYP450 2C9 Inhibitor	Non-substrate	0.9045	Non-inhibitor	0.5823	Non-inhibitor	0.5284	Inhibitor	0.6723
CYP450 2D6 Inhibitor	Non-substrate	0.9070	Non-inhibitor	0.9287	Non-inhibitor	0.8642	Non-inhibitor	0.8902
CYP450 2C19 Inhibitor	Non-substrate	0.9268	Non-inhibitor	0.9025	Non-inhibitor	0.6136	Non-inhibitor	0.6458
CYP450 3A4 Inhibitor	Non-substrate	0.9026	Inhibitor	0.6951	Non-inhibitor	0.5913	Inhibitor	0.5468
CYP Inhibitory Promiscuity Toxicity	Low CYP Inhibitory Promiscuity	0.8309	High CYP Inhibitory Promiscuity	0.5822	Low CYP Inhibitory Promiscuity	0.5415	High CYP Inhibitory Promiscuity	0.5251
AMES Toxicity	AMES toxic	0.7844	Non-AMES toxic	0.7220	Non-AMES toxic	0.5771	Non-AMES toxic	0.6568
Carcinogens	Non-carcinogens	0.9033	Non-carcinogens	0.9450	Non-carcinogens	0.8575	Non-carcinogens	0.8965
Biodegradation	Not ready	1.0000	Not ready	0.8672	Not ready	0.9740	Not ready	0.9707
Acute Oral Toxicity	III	0.7916	II	0.7348	III	0.5220	III	0.4607
Carcinogenicity (Three-class)	Non-required	0.6211	Non-required	0.6750	Non-required	0.4621	Non-required	0.5592
Rat Acute Toxicity (LD50, mol/kg)	2.1639		3.0200		2.7615		2.6564	

*Prob* probability (%).

## Discussion

The study analysed the extraction of bioactive compounds from *S. thinghirensis* WAE1 isolate using various organic solvents with varying polarities. The ethyl acetate solvent was found to be the most effective for extracting high yield and potent bioactive compounds against *S. pneumoniae* ATCC 49619. The study also found that chloroform extracts have poorer activity, suggesting they are suboptimal for extracting natural products. The large inhibition zone of the ethyl acetate extract indicates its potent antibacterial components against *S. pneumoniae*. This finding is consistent with literature studies indicating that ethyl acetate is the most effective solvent for extracting metabolites from fermentation medium and bioactive metabolites. Tangjitjaroenkun^32^ analyzed the extraction of bioactive compounds from *Streptomyces omiyaensis* SCH2 using an ethyl acetate solvent. The ethyl acetate extract yielded the highest amount of crude residue (1.86 g) and was found to be most effective for extracting potent bioactive compounds against test pathogens such as *Enterobacter cloacae*, *Klebsiella pneumoniae*, and *Bacillus subtilis* as indicated by the large inhibition zones in the disc diffusion assay. Kurnianto et al.^33^ analyzed the extraction of bioactive compounds from *Streptomyces* sp. AIA12 and AIA17 isolates using ethyl acetate solvent. Ethyl acetate was found to be most effective for extracting high yield and potent bioactive compounds against all test pathogens including *Pseudomonas aeruginosa* InaCC B52, *Staphylococcus aureus* ATCC 25923, *Bacillus cereus* ATCC 10876 and *Escherichia coli* ATCC 25922, as indicated by their Minimum Inhibitory Concentration and minimum bactericidal concentration values ranging from 2.5 to 0.31 mg/mL. The large inhibition zones produced by the ethyl acetate extracts, especially against *B. cereus*, indicates their potent antibacterial components.

Thin layer chromatography (TLC) was used to separate antibacterial agents into individual components. Three yellow spots were detected on the TLC plate, with the only spot with Rf 0.43 showing antibacterial activity against *S. pneumoniae*. The purification process through column chromatography showed active fractions against the bacterial pathogenic range 4 to 12, with the same retention factors (Rf) 0.43. These fractions were combined and assayed for identification as the active compound. The chemical characterization of the active extracted compound, characterized by UV-spectrum, FT-IR, 1H & 13C NMR, mass spectrum, and elemental analysis, is expected to be related to quercetin. Mothana et al.^34^ isolated three compounds from *Streptomyces* sp. 1S1 using column chromatography and thin layer chromatography (TLC). Compound 1 was identified as a trimyristin using NMR spectroscopy, while FTIR spectroscopy showed strong bands of ester and methylene functional groups. The ethyl acetate extract showed inhibitory activity against *S. pneumoniae, S. aureus*, *E. coli*, and *Bacillus subtilis*, indicating *Streptomyces* sp. 1S1 has biomedical potential through the production of antimicrobial metabolites against clinically relevant human pathogens. The combination of TLC, column chromatography, and NMR helped in structural elucidation.

Sensitivity testing showed *S. pneumoniae* to be resistant to some antibiotics like cefalexin and kanamycin, likely due to complex genetic resistance mechanisms. Penicillin, tetracycline and levofloxacin exhibited effectiveness. This study demonstrated that combinations of quercetin or its derivatives QCX with levofloxacin exhibited stronger anti-biofilm activity against *S. pneumoniae* than their individual MIC doses, indicative of synergistic interactions. Synergism arises when two drugs with distinct mechanisms simultaneously exert inhibitory effects, resulting in stronger bacterial killing than monotherapies. Performing assays at the lowest fractional inhibitory concentrations affirmed this synergism, with QCX-4 in combination with levofloxacin completely inhibiting biofilm formation at exceptionally low concentrations of just 0.25 μg/mL of each agent. Such synergistic combinations that achieve microbial growth suppression at far lower doses than monotherapy treatments have promising applications for combating antibiotic resistance by restoring drug efficacy. Additionally, quercetin and related natural products may offer safer alternatives to conventional antimicrobials. Overall, the findings suggest that combined regimens of flavonoids like quercetin with levofloxacin can enhance antibacterial function and may represent a clinically useful strategy for overcoming resistance in *S. pneumoniae* infections, warranting deeper investigation of these candidate combination therapies^35^.

Here, quercetin acts as a quorum sensing inhibitor to control population density while antibiotics induce cellular mortality. Levofloxacin inhibits DNA gyrase/topoisomerase, and quercetin exhibits multi-factorial activity against *S. pneumoniae* through reducing virulence factors, attenuating adherence, disrupting membranes and interfering with energy production^36,37^. Vipin et al.^9^ demonstrated that quercetin showed synergistic activity when combined with various antibiotics against multidrug-resistant *P. aeruginosa* strains isolated from catheter-associated urinary tract infections. Quercetin's ability to inhibit virulence factors regulated by quorum sensing in *P. aeruginosa* improves antibiotic penetration and bacterial killing, thus lowering antibiotic doses required for bacterial infection treatment.

Compound QCX-3 features a catechol-nickel core surrounded by hydroxylated aromatics, suggesting ability to interfere with metal homeostasis or electron transport pathways through metal chelation or reactive oxygen species generation^38^. Compound QCX-4 contains two chromone moieties linked by phenylene and substituted with four boron-containing dioxaborole groups. This highly substituted boron structure gives exceptionally high potential for multi-target interactions disrupting membranes/cell walls^39^. These structures enable multivalent binding to various cellular targets through their diverse functional groups. This multi-targeted multi-mechanistic strategy enhances antimicrobial potency and reduces resistance development potential, emphasizing the value of combining natural products with different mechanisms of action to address antibiotic resistance challenges^40^. Mutlu Gençkal et al.^38^ investigated that quercetin has antioxidant, anti-inflammatory, and antimicrobial properties when used alone or in complexes with metal ions. They found that Ni-quercetin complexes can enhance antibacterial activity due to increased lipophilic nature and cellular uptake. Temel et al.^22^ studied the antibacterial properties of novel boron and quercetin-containing ligands (B1 and B2). Boron compounds have been shown to be effective against both standard and multidrug-resistant bacteria due to their ability to disrupt enzymes. Quercetin, a natural flavonoid compound, has also shown antibacterial activity. By synthesizing hybrid boron-quercetin ligands, they aimed to develop new agents with synergistic activity against drug-resistant pathogens. Their evaluation of B1 and B2's antibiofilm and anti-quorum sensing effects provides insight into their potential as alternatives for combating microbial resistance.

The main mechanism by which quercetin inhibits biofilm development is through blocking the transpeptidase activity of SrtA. This, in turn, prevents the proper anchoring of NanB to the bacterial cell wall, disrupting the biofilm formation process^41^. Bacterial NAs are referred to as sialidases and have been found to reside in the respiratory tract, the gastrointestinal tract, the reproductive tract and the oral cavity. *Streptococcus pneumoniae* produces three sialidases (NanA, NanB and NanC), which desialylate host cell surfaces for bacterial adhesion. NAs contributes to mucosal colonisation, as it is involved in biofilm formation and contributes to infection by promoting pro-inflammatory responses^42^. Wang et al.^41^ conducted a study on the inhibition of *S. pneumoniae* sortase A (Spn-srtA) by the flavonoid quercetin. They found that quercetin competitively inhibited Spn-srtA activity in a dose-dependent manner, and that it occupied the substrate binding channel of Spn-srtA, hindering the binding of the bacterial cell wall sorting signal LPXTG. This suggests quercetin treatment could impair the attachment of pneumococcal virulence factors like neuraminidase B (NanB), which is essential for proper localization to the cell wall. The study also found quercetin significantly reduced biofilm mass in a NanB-dependent manner.

The computational analysis provided valuable insights by predicting cooperative relationships between glmM, nanA and sodA, particularly their involvement in cell envelope functions, based on multiple genetic and physical interaction datasets. While these genes were initially annotated to roles in peptidoglycan synthesis, sialic acid catabolism and oxidative defences, their links to central metabolism and stress response genes suggest more wide-ranging functions in the bacterium. Significant, the findings propose that compounds like Quercetin, levofloxacin, QCX-3 and QCX-4 may inhibit bacterial growth through interaction with these genes or their partners, representing potential leads for novel antibacterial therapeutics targeting the predicted networks without harming the host. Sudhakar et al.^43^ analysed the transcriptional regulatory response network of *Streptococcus* mutans biofilms after treatment with biofilm inhibitor carolacton. They used the Trend Correlation method to infer a co-expression network of 8284 gene–gene relationships and overlaid transcription factor binding site information to construct a transcriptional regulatory response network (TRRN) of 329 putative interactions. The sub-networks were modulated by regulators involved in pathways like pyrimidine biosynthesis and glutamine metabolism, as well as two component system response regulators. The predicted networks suggest that compounds inhibiting bacterial growth through interaction with these genes or their partners could represent potential therapeutics targeting pathogenic bacteria without harming the host.

The computational predictions provide insights into potential mechanisms of action and biological activities of these compounds but require experimental validation. Levofloxacin's predicted inhibition of critical bacterial processes involved in DNA maintenance and cell wall/membrane synthesis. For quercetin, the high probability predictions against essential bacterial functions related to metabolism, signalling and defences provide rationale to evaluate its antibacterial potential. QCX-3's predicted inhibition of key bacterial functions through multiple mechanisms supports evaluation of its anti-streptococcal effects upon experimental validation, along with safety assessment given its multifunctional profile. QCX-4's predicted inhibition of critical pathways involved in signalling and inhibition of bacterial growth through affecting on histidine kinase, fatty acid synthase and broad kinase inhibition, provides plausible mechanisms for its antibacterial ability and rationale for development as a novel anti-infective, particularly through combinatorial approaches. Amalini and Afidah^44^ conducted a study using in-silico methods, including PASS online, to analyse the bioactivities of secondary metabolites from *Ranunculus japonicus*. They identified compounds with high antioxidant potential, including 3-Methylquercetin. They determined their bioactivity using way2drug, which uses PASS to predict potential mechanisms of action and biological activities. 3-Methylquercetin was predicted to inhibit essential bacterial functions.

In-silico predictions suggest that levofloxacin and quercetin have potential for treating *S. pneumoniae* infections, regarding to their ADMET profiles. Both compounds show favorable absorption and distribution properties and potential CYP inhibition. QCX-3 and QCX-4 demonstrate the most promising profiles, with favorable permeability, distribution, low metabolism, and minimal toxicity risks. Hasan et al.^45^ conducted a study on Quercetin's ADMET properties and toxicity evaluation. The toxicity was evaluated using admetSAR online server, revealing no mutagenic or carcinogenic properties and lower rat acute toxicity. This indicated Quercetin's good safety profile. The study also evaluated Quercetin's molecular docking simulation against targets like mycolic acid cyclopropane synthase for its anti-tuberculosis activity. The higher docking score compared to standard drugs suggested Quercetin's potential as an antibacterial agent. This comprehensive analysis provided valuable insights into Quercetin's safety profile and potential antibacterial agent for conditions like tuberculosis.

## Conclusion

This study highlights the potential of quercetin complexes as promising adjuvants to conventional levofloxacin in the treatment of *S. pneumoniae* infections. Through coordinated multi-target inhibition of both bacterial resistance and virulence factors, QCX-3 and QCX-4 were shown to synergistically enhance the antibacterial activity and bactericidal kinetics of levofloxacin both in-vitro and mechanistically through molecular interactions with key enzymes SrtA and sialidase. Importantly, combination therapy more effectively inhibited biofilm formation compared to single agents, indicating these quercetin derivatives overcome a critical resistance mechanism in pneumococcal infections. With continued investigations optimizing these quercetin complexes and evaluating their efficacy and safety in clinically relevant models, this work presents an innovative therapeutic approach that can be further developed into clinically translatable anti-infectives to address the growing threat of antibiotic resistance in *S. pneumoniae* and other priority pathogens. Future investigations should assess these combinations in animal infection models to support their potential development as clinically effective anti-infectives.

### Supplementary Information

Supplementary Information.

## Data Availability

The datasets generated and analyzed during the current study are available from the corresponding author on reasonable request.
