# Supplementary material for: Therapeutic potential activity of quercetin complexes against Streptococcus pneumoniae
Source: Sci Rep. 2024 Jun 5;14:12876. doi: 10.1038/s41598-024-62782-w (PMC11150394; doi:10.1038/s41598-024-62782-w)
Supplement: Supplementary file 1 — Supplementary Information. [file 41598_2024_62782_MOESM1_ESM.docx]

**Table S1.** Antibacterial activity of the compound extracted with different solvents from *S. thinghirensis* WAE1.

| Solvent | Yield of crude extract (mg) | Inhibition Zone (mm) |
| --- | --- | --- |
| Acetone | 210 | 21.41 ± 0.15 |
| Chloroform | 72 | 11.72 ± 0.45 |
| Ethyl acetate | 840 | 34.85 ± 0.71 |
| Methanol | 450 | 27.45 ± 0.11 |
| n-Butanol | 81 | 18.57 ± 0.29 |
| n-Hexane | 26 | 16.32 ± 0.32 |

**Table S2.** Antibacterial activity of the crude extract’s purified spots.

| Spots (R_f_ value) | 0.43 | 0.56 | 0.72 |
| --- | --- | --- | --- |
| Inhibition Zone (mm) | 26.32 | 0 | 0 |

**Table S3.** Antibacterial activity of the column fractions.

| Fractions | 1 | 2 | 3 | 4 | 5 | 6 | 7 | 8 | 9 | 10 | 11 | 12 | 13 | 14 |
| --- | --- | --- | --- | --- | --- | --- | --- | --- | --- | --- | --- | --- | --- | --- |
| inhibition Zone (mm) | 0 | 0 | 0 | 8.1 | 11.4 | 12.6 | 13.2 | 14.3 | 21.9 | 17.1 | 11.7 | 9.3 | 0 | 0 |

**Table S4.** Sensitivity of antibiotics against *S. pneumoniae*.

| Antibiotic group | Β-lactam | Cephalosporin | Tetracycline | Aminoglycoside | Fluoroquinolone |
| --- | --- | --- | --- | --- | --- |
| Type of antibiotic | **Penicillin (10 µg/mL)** | **Cefalexin (30 µg/mL)** | **Tetracycline (10 µg/mL)** | **Kanamycin (10 µg /mL)** | **Levofloxacin (15 µg /mL)** |
| Sensitivity | S | R | S | R | S |

S: susceptible; R: resistance

**Table S5.** The MICs of susceptible antibiotics, quercetin and QCX compounds.

| Compounds | Penicillin | Tetracycline | Levofloxacin | Quercetin | QCX-1 | QCX-2 | QCX-3 | QCX-4 | QCX-5 | QCX-6 |
| --- | --- | --- | --- | --- | --- | --- | --- | --- | --- | --- |
| MIC (µg/mL) | 4 | 8 | 2 | 128 | 16 | 4 | 2 | 1 | 2 | 4 |

**Legends**

**Table S1.** Antibacterial activity of the compound extracted with different solvents from *S. thinghirensis* WAE1.

**Table S2.** Antibacterial activity of the crude extract’s purified spots.

**Table S3.** Antibacterial activity of the column fractions.

**Table S4.** Sensitivity of antibiotics against *S. pneumoniae.*

**Table S5.** The MICs of susceptible antibiotics, quercetin and QCX compounds.
